# Focal salvage therapy for local prostate cancer recurrences after primary radiotherapy: a comprehensive review

**DOI:** 10.1007/s00345-016-1811-9

**Published:** 2016-03-24

**Authors:** D. A. Smit Duijzentkunst, M. Peters, J. R. N. van der Voort van Zyp, M. A. Moerland, M. van Vulpen

**Affiliations:** Department of Radiation Oncology, University Medical Centre Utrecht, Q.02.2.314, Heidelberglaan 100, 3584 CX Utrecht, The Netherlands

**Keywords:** Prostate cancer, Radiorecurrent disease, Focal salvage, Iodine-125 brachytherapy, Cryotherapy, HIFU, Review

## Abstract

**Background/Aim:**

Patients with locally recurrent prostate cancer after primary radiotherapy can be eligible for salvage treatment. Whole-gland salvage techniques carry a high risk of toxicity. A *focal* salvage approach might reduce the risk of adverse events while maintaining cancer control in carefully selected patients. The aim of this review was to evaluate current literature to assess whether focal salvage leads to a comparable or favourable recurrence rate and less toxicity compared to whole-gland salvage.

**Methods:**

A literature search was performed using PubMed, Embase and the Cochrane Library. A total of 3015 articles were screened and assessed for quality. Eight papers [on focal cryoablation (*n* = 3), brachytherapy (*n* = 3) and high-intensity focused ultrasound (*n* = 2)] were used to report outcomes.

**Results:**

One-, 2-, 3- and 5-year biochemical disease-free survival (BDFS) ranges for focal salvage are, respectively, 69–100, 49–100, 50–91 and 46.5–54.5 %. Severe genitourinary, gastrointestinal and sexual function toxicity rates are 0–33.3 %. One study directly compares focal to whole-gland salvage cryotherapy, showing 5-year BDFS of, respectively, 54.4 and 86.5 % with lower toxicity rates for focal salvage patients.

**Conclusion:**

Provisional data suggest that BDFS rates of focal salvage are in line with those of whole-gland approaches. There is evidence that focal salvage could decrease severe toxicity and preserve erectile function.

**Electronic supplementary material:**

The online version of this article (doi:10.1007/s00345-016-1811-9) contains supplementary material, which is available to authorized users.

## Introduction

Prostate cancer (PCa) patients primarily treated with external beam radiotherapy (EBRT) or brachytherapy (BT) are at risk of a recurrence, depending on pretreatment characteristics. Intermediate- and high-risk groups can suffer from a biochemical recurrence in over 50 % of the cases after 10-year follow-up [1]. Subsequently, they are at risk of developing metastases and dying of PCa [2]. Up to 98 % of patients receive (palliative) androgen deprivation therapy (ADT) as treatment after a biochemical recurrence [3]. However, a substantial amount of patients harbour organ-confined disease eligible for a curative salvage procedure [4, 5], thereby preventing exposure of patients to the often severe side effects of ADT [6]. Salvage nowadays is usually performed using a whole-gland approach, which is accompanied by a high chance of severe gastrointestinal (GI), genitourinary (GU) and erectile toxicity due to previous radiation damage to surrounding organs at risk [4, 5]. Given the evidence from pathology studies that recurrences are frequently localised at the site of the primary largest (index) tumour [7, 8], a focal salvage approach, directed solely at the area containing recurrent tumour, might be a viable treatment option for patients with unifocal PCa recurrences without metastatic disease. This way, serious adverse events associated with whole-gland salvage might be prevented, while cancer control is maintained. The current literature regarding biochemical disease free survival (BDFS) and functional outcomes of focal salvage techniques for prostate cancer recurrences after primary radiotherapy is evaluated here.

## Materials and methods

### Literature search

**Fig. 1:**
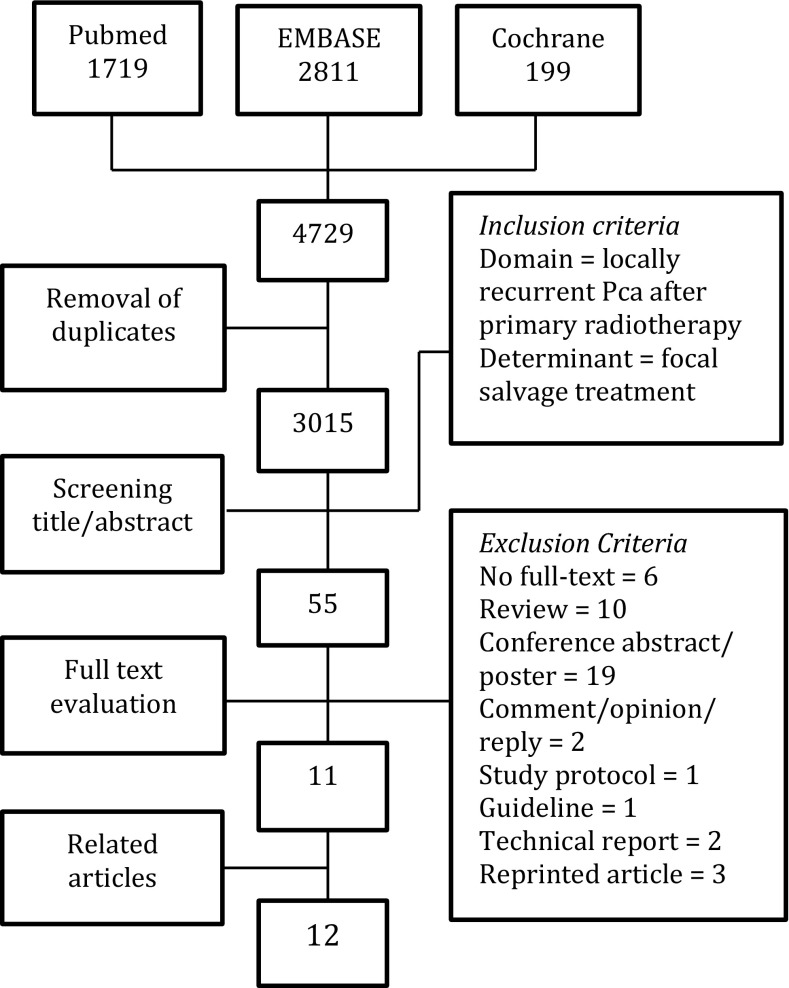
Flow chart

On 19 August 2015, a systematic literature search was performed in the PubMed, Embase and Cochrane Library databases. In order to create a sensitive search, the search syntax was build based on domain and determinant (Table 1). After removal of duplicates, 3015 papers were screened, selecting 55 articles with matching domain and determinant. Exclusion criteria are listed in the flow chart (Fig. 1). An additional reference search was performed, resulting in 12 studies for this review [9–20].

**Table 1:** Search syntax

All search terms (title/abstract) for PubMed, (ab,ti) for Embase and (:ti,ab,kw) for Cochrane			
1	Salvage	14	Local
2	Therapy	15	Focal
3	Treatment	16	Prostate
4	Rescue	17	Prostatic
5	Cryoablation	18	Cancer
6	Cryosurgery	19	Carcinoma
7	Cryotherapy	20	Adenocarcinoma
8	Ablation	21	Neoplasma
9	Brachytherapy	22	Recurrence
10	HIFU	23	Recurrences
11	‘high-intensity focused ultrasound’	24	Recurrent
12	Hemi	25	Relapse
13	Partial	26	Radiorecurrent
27	1 or 2 or 3 or 4 or 5 or 6 or 7 or 8 or 9 or 10 or 11		
28	12 or 13 or 14 or 15		
29	27 and 28		
30	16 or 17	#34 Search Results	
31	18 or 19 or 20 or 21	PubMed: 1719	
32	22 or 23 or 24 or 25 or 26	Embase: 2811	
33	30 and 31 and 32	Cochrane: 199	
34	29 and 33	Total: 4729

### Study selection

Studies were subjected to a critical appraisal based on an adaptation of the Dutch Cochrane Centre recommendations (ACROBAT-NRSI risk of bias assessment tool) [21]. Studies were graded (+), (±) or (−) on relevance (domain, determinant, outcome) and validity (selection, study population characteristics, exposure, primary outcome, secondary outcomes, follow-up and number of patients) [Table A1 (supplementary file)].

All studies were observational cohorts or case series with either prospective or retrospective data acquisition. Therefore, no study could be considered of high quality. Blinding was applied in none of the studies (not graded). However, the primary outcome (BDFS) is unlikely to be influenced by information bias, due to the objectivity of the failure definition (Phoenix, i.e. PSA-nadir + 2.0 ng/ml). On the contrary, the toxicity assessment is subjective to information bias and is graded in that way.

Confounding was not graded, since all studies except one were single-arm observational cohorts. The study by De Castro et al. [9] describes a two-armed cohort. Here, possible confounding factors are adequately described, but not corrected for in multivariable analysis.

Four studies describe case series ≤10 patients [12, 13, 19, 20]. These were valued as low quality and excluded.

## Results

### Search results

A total of eight studies were eligible for data extraction, describing focal salvage treatment with cryoablation (CA) (*n* = 3) [9, 14, 16], low-dose-rate brachytherapy (LDR-BT) (*n* = 3) [15, 17, 18] and high-intensity focused ultrasound (HIFU) (*n* = 2) [10, 11] in 278 patients. Table 2 shows study characteristics. Studies used a salvage approach on a focal lesion (quadrant ablation by Ahmed et al. [10] and clinical target volume of approximately 17 % by Hsu et al. [15]), one half of the prostate (hemi) or a partial approach (area not specified by Li et al. [16] and Eisenberg et al. [14]). Nguyen et al. [17] describe LDR-BT to the entire peripheral zone. In all studies, ADT use was discontinued at time of enrolment.

**Table 2:** Study characteristics and BDFS

Study	Salvage treatment	Patients	Primary treatment	Primary radiation dose/schedule	Age	Time between treatments	Diagnosis of focality	Number of biopsies	MRI sequences	Exclusion metastases
De Castro Abreu [9]	Cryo (hemi)	25	EBRT 11 (44); PB 8 (32); BT 5 (20); BT + EBRT 1 (4)	NA	71 (59–81)	8.3 (3–15)	TRUS/TRUS Biopsies	NA	NA	BS, pelvic CT/MRI if Gleason ≥ 7 or PSA ≥ 10
	Cryo (whole-gland)	25	EBRT 11 (44); PB 5 (20); BT 7 (28); BT + EBRT 2 (8)		73 (57–83)	6.3 (2–13)				
Ahmed [10]	HIFU (hemi 16, focal/quadrant 23)	39	EBRT 39 (100)	Median 64 Gray (range 50–74)	70 (±6.8)	6.5 (4–15)	MRI, TRUS (19)/TPM (20) biopsies	NA	1.5T: T2, DCE, DWI	pelvic MRI, BS, 18F–PET
Baco [11]	HIFU (hemi)	48	EBRT 46 (96); BT 2 (4)	Mean 72.5 Gray (range 64–78, sd 3.3)	68.8 (±6.0)	5.9 (±2.6)	MRI/TRUS biopsies	Mean 15 (range 7–60, sd 10)	1.5T/3T: T1/T2, DCE, DWI	BS, pelvic CT/MRI, 11C-PET (*n* = 27)
Eisenberg [14]^d^	Cryo (partial)	15	EBRT; EBRT + BT	NA	70 (58–86)	6 (±NA)	TRUS biopsies	16	NA	BS/CT
Hsu [15]	LDR-BT (partial)^e^	15	LDR-BT 15 (100)	>144 Gray	66.2 (±6.1)	5.7 (2.3–11)	MRI	NA	T2, MRSI	BS/CT
Li [16]	Cryo (partial)	91	BT 25 (27); EBRT 44 (48); BT + EBRT 3 (3); unknown 19 (21)	NA	71.1 (±7.2)	NA	NA	NA	NA	NA
Nguyen [17]	LDR-BT (peripheral zone)	25	EBRT 13 (48); BT 11 (44); EBRT + BT 1 (4)	Range 66–70.2	65 (56–82)	5.2 (2.5–12.8)	MRI	NA	NA	BS, pelvic CT/MRI
Peters [18]	LDR-BT (focal)	20	LDR-BT 7 (35); EBRT 6 (30); IMRT 7 (35)	70 Gray (6), 76 Gray (7)	69 (59–78)	6.6 (3.5–12)	MRI	Median 10 (range 6–13)	3T T1, T2, DCE, DWI	BS, pelvic CT/MRI, 18F-PET (*n* = 10)

Study	D’Amico Risk classification	Pretreatment PSA	Neoadjuvant ADT	Follow-up	BF definition	1 Year BDFS (%)	2 Year BDFS (%)	3 Year BDFS (%)	4 Year BDFS (%)	5 Year BDFS (%)
De Castro Abreu [9]	NA	7 (2.8–19.8) 2.8 (0.1–8.2)	9 (37)	31 (4–90)	Phoenix					54.4
		6 (1.8–23.8) 3.9 (0.1–12)	7 (28)	53 (12–92)	Phoenix					86.5
Ahmed [10]	6/13/15/5	19 (0.2–129) 3.3 (0.02–27.9)	NA (33)	17 (10–29)^b^	Phoenix	69	49			
Baco [11]	10/20/20/6	14.2 (+12.1) NA	11 (23)	16.3 (10.5–24.5)^b^	Phoenix (ASTRO, *n* = 2)	83	52			
Eisenberg [14]^d^	NA	NA 3.3 (0.28–8.96)	NA	18 (6–33)	ASTRO Phoenix	89 89	67 79	50 79		
Hsu [15]	11/4/0/0	7.4 (4.1–16.2) 3.5 (0.9–5.6)	4 (26.7)^a^	23.3 (NA)	ASTRO Phoenix	86.7 100	78.4 100	62.7 71.4		
Li [16]	NA	NA 4.8 (0–92.6)	32 (35.2)	15 (1–97)	Phoenix	95.3		72.4		46.5
Nguyen [17]	NA	7.45 (4.2–18.4) 5.5 (1.4–11.6)	1 (4)^a^	47 (14–75)	Phoenix	100^c^	91^c^	91^c^	70	
Peters [18]	5/3/12/0	12.9 (5.4–51) 4.7 (0.3–14.0)	8 (40)	36 (10–45)	Phoenix			60.0		

*ADT* androgen deprivation therapy, *ASTRO* American Society for Therapeutic Radiology and Oncology, *NA* not available, *TRUS* transrectal ultrasound, *BDFS* biochemical disease-free survival, *BF* biochemical failure, *EBRT* external beam radiotherapy, *PB* proton beam radiotherapy, *BS* bone scan, *HIFU* high-intensity focused ultrasound, *LDR-BT* low-dose-rate brachytherapy, *TPM* transperineal template prostate mapping (biopsies), *DCE* dynamic contrast enhanced, *DWI* diffusion-weighted imaging, *FDG-PET* fluorodeoxyglucose-positron emission tomography, *MRSI* MR spectroscopy imaging

Study: first author, year, (reference). Salvage treatment: modality (approach). Primary treatment: modality, *n* (%). Age: at salvage treatment, median (range) or mean (±sd). Time between primary and salvage treatment: years, median (range) or mean (±sd). D’Amico Risk Classification: at primary diagnosis, low/Intermediate/High/Unknown, *n*. Pretreatment PSA: preradiotherapy and presalvage, median (range) or mean (±sd), ng/ml

Neoadjuvant ADT: before salvage or ^a^ at time of primary treatment, *n* (%). Follow-up: in months: median (range) or ^b^ (interquartile range). ^c^ Estimation derived from Kaplan–Meier curve. ^d^ Baseline characteristics of initial 19 patients, of which 15 used for follow-up data. ^e^ One (1) patient received 40 Gy/20 fractions IMRT additional to LDR-BT

### Diagnosis of focality

All recurrences were initially detected by PSA measurement (biochemical failure), verified with prostate biopsies. The diagnostic modalities used to determine the recurrence location differed. Available specifics are described here.

Five studies used magnetic resonance imaging (MRI). Ahmed et al. [10] describe the use of a multiparametric approach: T2-weighted (T2 W), diffusion-weighted (DW) and dynamic contrast-enhanced (DCE) 1.5-Tesla (T) MRI. Results were combined with transrectal ultrasound (TRUS)-guided or transperineal template prostate mapping (TPM) biopsies (*n* = 19 and *n* = 20, respectively). Baco et al. [11] describe the use of T2 W, DW and DCE 3T MRI in 27 and T2 W and DW 1.5T MRI in 21 patients. Imaging results were verified a mean 15 cores TRUS-guided biopsies (sd 10, range 7–60). Peters et al. [18] describe the use of T1 W, T2 W, DWI and DCE 3T MRI, verified with systematic transrectal biopsies (median 10 cores, range 6–13). Sequences are not specified in the studies of Nguyen et al. [17] and Hsu et al. [15], though the latter did perform MR spectroscopy and acquired TRUS-guided biopsy confirmation of disease. Both do not define the amount of biopsy cores taken.

De Castro Abreu et al. [9] treated the tumour volume based on both systematic and lesion-targeted TRUS-guided biopsies and hypo-echogenic lesions on TRUS. Eisenberg et al. [14] treated based on 16-core sample TRUS-guided biopsy. Li et al. [16], using the Cryo On-Line Data (COLD) registry, do not describe any diagnostic modality used to define the location of the recurrence or (PSA-based) selection method.

### Exclusion of metastatic disease

Three studies used positron emission tomography (PET) in excluding metastatic disease (Ahmed et al. [10], Baco et al. [11] and Peters et al. [18]). Ahmed et al. [10] used a combination of pelvic MRI, a radioisotope bone scan and 18-fluorodeoxyglucose (FDG) PET/CT in all patients for the assessment of metastatic disease (bone and lymph node). Baco et al. [11] used a combination of bone scan and pelvic CT or MRI. 11C-Choline PET was used in 27 of 42 patients. Peters et al. also use a bone scan and pelvis CT or MRI, but used 18F-Choline PET in 10 of the 20 patients.

### Biochemical disease-free survival

Results on BDFS are also shown in Table 2. Biochemical failure was defined according to the Phoenix (PSA-nadir + 2 ng/ml) or ASTRO definition (three consecutive post-nadir rises in PSA, with the moment of failure backdated between the nadir and the first rise). BDFS ranges at 1, 2, 3 and 5 years are, respectively, 69–100, 49–100, 50–91 and 46.5–54.4 %. The studies with an MRI-based assessment of focal disease had BDFS of 49–100 % up to 2–3 years compared to 72.4–79 % for non-MRI-based focal salvage series. The only 5-year Kaplan–Meier estimates come from the study by de Castro-Abreu et al. [9] and Li et al. [16], who do not use MRI for intraprostatic disease assessment or PET for exclusion of metastatic disease (de Castro-Abreu et al. [9]) or do not specify the assessment (Li et al. [16]). They provide BDFS of 46.5 % (Li et al. [16]) and 54.4 % (de Castro-Abreu et al. [9]).

### Toxicity

Toxicity results are shown in Table 3 for various measurement methods. CTCAE grades indicate (1) toxicity without (the need for) intervention, (2) requiring medication, (3) requiring inpatient or outpatient surgical intervention, (4) requiring ICU admission and (5) death. No grade 4 or 5 toxicity was reported. Higher IPSS (prostate symptoms), lower IIEF (erectile function) and lower QLQ C-30 (quality of life) indicate deterioration. In addition, Ahmed et al. [10] reported surgical complications according to the modified Clavien system, showing the need for intervention under local (grade 3a) or general (grade 3b) anaesthesia in, respectively, 1 (3 %) and 9 (23 %) patients. Nguyen et al. [17] reported outcomes according to the Radiation Therapy Oncology Group/Late Effects Normal Tissue Task Force criteria, showing rectal bleeding in 2 (8 %), urethral stricture in 1 (4 %), periprostatic abscess in 1 (4 %) and prostate–rectal fistula in 3 (12 %) patients.

**Table 3:** Toxicity

Study	New CTCAE GU Toxicity grade ≥2	New CTCAE GI Toxicity grade ≥2	New CTCAE SF Toxicity grade ≥2
Hsu	4 (26.7)	0 (0)	4 (26.7)
Peters	6 (33.3)	0 (0)	0 (0)

Study	New Clavien Toxicity grade ≥2	New late RTOG Toxicity grade ≥3	
Ahmed	10 (30)		
Nguyen		7 (28)	

Study	IPSS before/at last FU	IIEF before/at last FU	QLQ C-30 before/at last FU
Ahmed	10.1/13	18/13^a^	
Baco	7.1/8.6	11.2/7.0	35.7/36.8

Study	New incontinence	New urethral toxicity	Potency before/after salvage
De Castro Abreu (focal vs. total)	0 versus 3 (0 vs. 12)	0 versus 1 (0 vs. 4)	7/2 versus 4/0 (28/8 vs. 16/0)
Eisenberg	1 (6.7)	2 (13.3)	
Li	5 (5.5)	3 (3.3)	20/10 (21.2/11)

*CTCAE* Common Terminology Criterial for Adverse Events, *GU* genitourinary, *GI* gastrointestinal, *SF* sexual function, *RTOG* radiation therapy oncology group, *IPSS* International Prostate Symptoms Score, *IIEF* International Index of Erectile Function, *QLQ C30* European Organisation for the Research and Treatment of Cancer Quality of Life Questionnaire, *FU* follow-up

Toxicity rates in numbers (%)

Questionnaire results: mean or ^a^ median. Urethral toxicity: strictures, ulcers and recto-urethral fistulae formation

Both Hsu et al. [15] and Peters et al. [18] report no new ≥grade 2 GI toxicity in the late phase (>3–6 months). Hsu et al. [15] report on five patients with ≥grade 2 GU toxicity requiring medication. Furthermore, two patients developed new medication-resistant erectile dysfunction (ED, grade 3) and two patients medication-responsive ED. Peters et al. [18] report one patient (5 %) with a grade 3 urethral stricture, requiring endoscopic incision. One patients suffered from radiation cystitis grade 2, managed with hyperbaric oxygen therapy. Five more patients had grade 2 urinary frequency, managed with medication. No new erectile dysfunction (ED) was seen in the five previously potent patients. Furthermore, a significant decrease in the EORTC-PR25 urinary symptoms quality-of-life (QoL) domain after a median of 3 years was observed.

Functional deterioration was also observed in symptom scales used by Baco et al. [11] and Ahmed et al. [10]. An International Prostate Symptom Score (IPSS) increase from 7.1 to 8.6 (*p* = 0.13) and 10.1 to 13 (no *p* value) was seen, respectively. Furthermore, International Index of Erectile Function (IIEF) decreased from mean 11.2 to 8 (*p* < 0.001) and median 18 to 13 (no *p* value), respectively. Baco et al. [11] do not notice a significant decrease in EORTC QLQ-C30 score: 35.7–36.8 (*p* = 0.22).

Eisenberg et al. [14] describe the occurrence of one mild stress incontinence (grade not given), one urethral stricture requiring dilation and one prostatic urethral ulcer managed with suprapubic catheter drainage. Two out of five patients remained potent. Li et al. [16] describe 5 (5.5 %) patients requiring pad-use at 12 months and 3 (3.3 %) recto-urethral fistulas. Half of the patients retained potency, although medication was not specified.

A subdivision in more focally targeted ablation and studies using MRI-assessment for recurrences was not made due to (most importantly) the unavailability of salvage extent.

## Discussion

### Comparing focal to whole-gland salvage

The aim of this study was to assess whether focal salvage for local prostate cancer recurrences after primary radiotherapy leads to a comparable or favourable recurrence rate and less toxicity compared to whole-gland salvage. De Castro Abreu et al. [9] present the only study comparing focal to whole-gland salvage cryotherapy. Recurrences were not verified by MR imaging. BF occurred in 32 % (focal) and 12 % (whole-gland), resulting in 5-year Kaplan–Meier BDFS estimates of, respectively, 54.4 and 86.5 %. This could be an indication that focal salvage patients are undertreated or that the relation is confounded by other factors related to BF. Since recurrences were not assessed by MRI or TPM biopsies, focal salvage patients might have been under assessed. Furthermore, the comparison indicates that focal salvage might be less toxic: no focal salvage patients developed incontinence or recto-urethral fistula versus 3 and 1, respectively. Furthermore, two focal salvage patients retained potency, versus none in the total salvage group. However, these numbers were small and statistical significance was not achieved. This comparison between the two ablation methods is hampered by differences in primary radiation schedules/modalities, the extend of focal salvage and differences in patient characteristics.

Whole-gland salvage outcomes are shown in Table 4. Systematic reviews on salvage therapies for radiorecurrent PCa [5, 22, 23] and the European Association of Urology (EAU) guideline [24] were searched to select the three largest studies of the four common salvage therapies (salvage radical prostatectomy (SRP) [25–27], BT [28–30], CA [31–33] and HIFU [34–36]). These publications do not provide recommendations on the type of whole-gland salvage treatment to use.

**Table 4:** Overview of whole-gland salvage outcomes

References	Salvage treatment	Patients (*n*)	Age	Presalvage PSA	Neoadjuvant ADT	Failure definition	Time period	FFS	Incontinence	Wound infection	BNS	Urinary extravasation	Erectile dysfuncton
Chade [25]	SRP	404	65 (60–69)^a^	4.5 (2.5–7.4)^a^		0.2 or 0.1 and rising	5 Y	48					
Paparel [26]	SRP	146	65 (61–69)^a^	5.1 (2.7–8.9)^a^		≥0.2 or start ADT	5 Y	54					
Ward [27]	SRP	138	65.1 (±6.1)	8.9 (±13.5)	23^c^	≥0.4 or clinical	5 Y	58	48	4	22	15	

Study	Salvage treatment	Patients (*n*)	Age	Pretreatment PSA	Neoadjuvant ADT	Failure definition	Time period	FFS	Incontinence	Grade ≥2 GU toxicity	Grade ≥2 GI toxicity	Recto-urethral fistula	Erectile dysfuncton
Chen [28]	HDR-BT	52	67.5 (53.9–81.4)	9.3 (1.2–58)	46	Phoenix or clinical	5 Y	51		56	2		35
Grado [29]	LDR-BT	49^b^	73.3 (52.9–86.9)	5.6 (1.5–79.1)	24^c^	Two successive rises post-nadir or clinical	5 Y	34	6.1	24.5	6.2		
Burri [30]	LDR-BT	37	70 (51–79)	5.6 (1.7–35.0)	84	Phoenix	5 Y	64.5	5.4	38	8	1	23 of 27

Study	Salvage treatment	Patients (*n*)	Age	Pretreatment PSA	Neoadjuvant ADT	Failure definition	Time period	FFS	Incontinence	Post-cryo TURP	Acute Retention	Recto-urethral fistula	Erectile dysfuncton
Spiess [31]	Cryo	450	64.1 (50.7–78.2)^d^	17.8 (1.3–157.1)^d^	54.6	>0.5	3.4 Y^e^	34^e^					
Pisters [32]	Cryo	279	70 (±7.1)	7.6 (±8.2)	50.9	Phoenix	5 Y	54.5	4.7	3.2		1.2	69.2
Ng [33]	Cryo	187	70.9 (53.6–81.7)	4.9 (0–36.4)	71	Phoenix or clinical	5 Y	56/29/14^f^	39.6	2.7	21.4	2.1	

Study	Salvage treatment	Patients (*n*)	Age	Pretreatment PSA	Neoadjuvant ADT	Failure definition	Time period	FFS	Incontinence	Anal Incontinence	BNS	Recto-urethral fistula	Urinary Sphincter Implantation
Murat [34]	HIFU	167	68.4 (±6.2)	6.8(±7.8)	56.8	Phoenix	3 Y	53/42/25^g^	49.7	1.2	19.8	3	10.8
Gelet [35]	HIFU	71	67 (±5.9)	7.7 (±8.1)		Biochemical or clinical	2.5 Y	38	35.2		16.9	5.6	5.6
Berge [36]	HIFU	57	67.4 (55–78)	5.8 (0.2–19)		Phoenix or start ADT	17.5 M^h^	67^h^				5.3	

*FFS* freedom of failure survival, *SRP* salvage radical prostatectomy, *H/LDR-BT* high-/low-dose-rate brachytherapy, *HIFU* high-intensity focused ultrasound, *BNS* bladder neck stenosis, *GU* genitourinary, *GI* gastrointestinal. Age: median (range) or mean (±sd). Pretreatment PSA: median (range) or mean (±sd). BF definition: PSA in ng/ml; clinical failure: evidence of disease clinically, radiologically or histopathologically. Neoadjuvant ADT, FFS and all toxicity in % unless otherwise specified. Toxicity grades according to the Common Terminology Criteria for Adverse Events

^a^Interquartile range. ^b^ 4 (8 %) also had previous (partial) prostatectomy. ^c^ Of which part had orchiectomy. ^d^ At initial diagnosis. ^e^ 66 % failure after median FU of 3.4 years. ^f^ For patients with PSA-nadir ≤4/4–10/≥10. ^g^ For D’Amico low-/intermediate-/high-risk patients. ^h^ 33 % failure after median FU of 17.5 months

Focal salvage BDFS rates (1, 2, 3, 5 years, respectively, 69–100, 49–100, 50–72.4 and 46.5–54.4 %) are in line with BDFS outcomes reported for whole-gland salvage. The largest SRP and CA studies show 5-year freedom of failure survival (FFS) rates of, respectively, 48–58 and 54.5–56 %.

Notable results outside these ranges are reported by Spiess et al. [31], describing 450 whole-gland salvage CA patients, with 66 % failure after median follow-up of 3.4 years. Grado et al. [29] describe 49 patients treated with BT, with a 5-year FFS of 34 %. Contrary to these results, Burri et al. [30] describe 37 patients treated with BT, with a 5-year FFS of 64.5 %.

In addition to reported results, Wenske et al. [37] describe a cohort of 55 focal CA patients after primary radiotherapy (80 %) or CA (20 %). There is no stratification for primary therapy. Reported 5-year BDFS (Phoenix definition) is 47 %. Bladder outlet obstruction occurred in 1.8 % and fistula formation in 5.5 % of patients.

Toxicity of focal approaches is comparable to or favourable compared to whole-gland salvage. Fistula rates are low across all studies. Notable results are reported by Hsu et al. [15] describing 15 focal salvage BT patients. There was no new GI toxicity and no new incontinence. Potency (medication assisted) was preserved in 13/15. Peters et al. [18] report preserved potency in the five previously potent patients. Potency is sparsely reported in SRP cohorts. From the assessed reviews, two SRP studies were retrieved describing SF. Masterson et al. [38] report preserved (medication assisted) potency in 6/40 (15 %), Boris et al. [39] in 2/10 (20 %).

### Limitations

#### Recurrent disease

Biochemical recurrences in prostate cancer often seem to stem from a localised process. Data on the exact location are scarce, but it is suggested that the tumour often recurs at the site of the primary dominant or ‘index lesion’ [7, 8, 40, 41]. The evidence is not unanimous, however, with tumour regrowth frequently occurring multifocally in some studies, although organ-confined and unifocal disease remains being observed [42, 43]. Also, radical prostatectomy or TPM biopsies are sometimes not used as the reference standard [7, 40], thereby possibly giving an overestimation of local recurrences. The studies which use pathology as the reference standard usually identify less unifocal disease localisation [42, 43]. However, in these studies patients were often treated with lower doses of radiation than with current dose escalation. A recent large analysis by Zumsteg et al. [1] in which 2.694 patients were treated with IMRT with a total dose ≥79.2 Gy (maximal 85.6 Gy) shows that biochemical recurrences are still common. Estimated 8-year recurrence rates of 9.7, 22.7 and 43.9 % for low, intermediate and high National Cancer Comprehensive Network (NCCN) risk groups was observed. It might be that with these dose schedules, secondary tumour foci are increasingly successfully treated. This could mean that radiorecurrent disease is shifting towards the index lesion and theoretically more patients in the future can be eligible for focal salvage. In addition, even though cancer control rates are increasing with further dose escalation, ADT use and enhanced patient selection, there will be an increase in absolute numbers of patients with biochemical recurrent disease because more patients are primarily treated with radiotherapy. The stage migration to lower risk disease due to PSA screening could lead to more recurrences originating from increasingly lower risk disease, which is possibly more often localised and unifocal.

### Study limitations

Reporting outcomes and comparing studies is significantly limited by several factors. The primary limitation is the lack of randomised controlled trials. All studies found are observational cohorts or case series. Selection of a more favourable or motivated patient population is a possibility in these focal salvage groups, thereby possibly biasing the comparison between focal and whole-gland salvage and between focal salvage modalities in terms of cancer control and toxicity. Furthermore, the lack of blinding in all studies could have biased especially toxicity assessment, both on patient and physician level.

Secondly, a straightforward comparison of studies is difficult. There is no international consensus on the definition of failure. Although there is a tendency to report BDFS according to the Phoenix definition (used in all focal salvage studies), there is a wide variety of failure definitions, including combined biochemical and clinical (physical, radiological, histopathological) proof of disease. Gelet et al. [35] report a 73 % negative biopsy rate at 30 months; however, combining biopsy results with biochemical results and need for ADT, the disease-free rate drops to 38 %. In reporting toxicity outcomes, there is little consistency as well.

In addition, diagnostic modalities for the assessment of recurrences and the exclusion of metastases vary. Only Ahmed et al. [10], Peters et al. [18] and Baco et al. [11] use PET in all, 10 and 27 patients, respectively, to exclude metastatic disease. PET has increased accuracy in assessing lymph node and distant metastases over technetium-99 scintigraphy and/or CT/MRI [44–46]. This could have contributed to more favourable outcomes in terms of cancer control and could possibly lead to a further increase in the future if these modalities become the diagnostic standard. Also, new biopsy techniques could lead to a further increase in the assessment of a focal recurrence, without missing possible significant multifocal recurrent disease. TPM biopsies could lead to increased accuracy over systematic TRUS-guided biopsies alone, while MRI-guided biopsies might decrease the detection of insignificant disease further [47, 48]. However, TPM biopsies were only adopted by Ahmed et al. [10], and MRI-guided biopsies by Baco et al. [11]. The other studies used different TRUS-guided schedules, thereby possibly undertreating the prostate with a focal salvage approach.

Finally, study populations are relatively small, limiting power, and follow-up is relatively short, limiting the number of outcome events and thereby an accurate estimation of BDFS proportions and late toxicity.

### Future trials

When considering trials for salvage modalities, willingness for randomisation is essential. This has been extremely problematic in a randomised study comparing prostatectomy and brachytherapy [49]. This accrual problem was also present in the PIVOT trial, in which only 15 % of patients were randomised [50]. Increasing favourable data from focal salvage studies (e.g. from the recently started FORECAST trial [Focal Recurrent Assessment and Salvage Treatment for Radiorecurrent Prostate Cancer] [51]) might further decrease the willingness for randomisation in potential future head to head salvage trials.

A solution can possibly be found in the cohort multiple randomised controlled trial (cmRCT) design [52]. In this study design, all patients with radiorecurrent disease (or all prostate cancer patients) in a centre would be included into a cohort. With consent, these patients can in the future be randomised into experimental and control groups when a new treatment becomes available, providing the standard of care to the control group and, after additional consent, a new modality to the experimental group. This could provide unbiased comparisons between salvage procedures and possibly even between salvage ablation modalities, without the need for substantial resources to achieve sufficient accrual.

## Conclusion

In this review of studies on focal salvage therapies, provisional data suggest that BDFS rates after focal salvage are in line with those of whole-gland approaches. There is evidence that focal salvage could decrease severe toxicity and preserve erectile function. Based on these results, focal salvage can be considered a viable option for unifocal prostate cancer recurrences after primary radiotherapy in properly selected patients. For further research, there is a great need for randomised controlled trials comparing salvage ablation methods and possibly even modalities. These trials would need to be uniform regarding patient selection and in outcome assessment and reporting. Lastly, relevant endpoint (mortality) assessed after sufficient follow-up are preferred over proxy outcomes such as biochemical failure.

## Electronic supplementary material

Below is the link to the electronic supplementary material.

Supplementary material 1 (XLSX 20 kb)
